# Intraoperative Dental Injury in a Neurosurgical Patient: Concerns for the Anesthesiologist

**DOI:** 10.7759/cureus.31268

**Published:** 2022-11-08

**Authors:** Stuti Bhamri, Sandeep Dey, Mukesh Gupta, Barkha Bindu, Manish Arora

**Affiliations:** 1 Neuroanesthesiology and Neurocritical Care, Paras Hospital, Gurugram, IND

**Keywords:** motor evoked potential, transesophageal echocardiography, sitting position, teeth subluxation, dental injury

## Abstract

Anesthesia-related oropharyngeal injuries are known to occur. Risk factors for intraoperative dental injuries include difficult intubation, use of transesophageal echocardiography (TEE) probe, motor-evoked potential (MEP) monitoring, poor dental hygiene, etc. Our patient was a case of a thalamic cavernoma who underwent craniotomy in a sitting position with the neck flexed along with MEP and TEE monitoring. At the end of the surgery, the lower three incisors were found to be subluxated. The subluxated teeth were stabilized using a 2-0 Ethilon suture in the operation room. Immediate dental consultation was sought postoperatively. Sitting position surgeries with associated neck flexion, simultaneous, advanced monitoring techniques like TEE and MEP, poor dental condition, and the use of hard bite blocks can predispose patients to dental injury. Preoperative dental evaluation and explanation of neuromonitoring-associated injuries can be beneficial.

## Introduction

Dental injuries are a common reason for medicolegal suits against anesthesiologists [1]. The incidence of intraoperative dental injuries varies from 0.07-12.1% in retrospective studies [2,3]. The most commonly reported injuries were enamel fractures, subluxated teeth, tooth avulsion, and crown or root fractures. Around 14% of these injuries go unnoticed and are reported later by patients [2]. Here, we present an unusual case of dental injury in a patient who underwent excision of thalamic cavernoma in a sitting position with intraoperative transesophageal echocardiography and neuromonitoring. Motor-evoked potential monitoring-induced bite injuries are well known. However, techniques like a partial neuromuscular blockade to avoid such bite injuries are not routinely practiced owing to various reasons. Written consent to publish this case report was obtained from the patient.

## Case presentation

A 50-year-old female of the American Society of Anesthesiologists (ASA) physical status I, weighing 56 kg, was posted for surgical excision of a thalamic cavernoma in a sitting position with transcranial electrical motor-evoked potential (MEP) monitoring. A preoperative physical examination revealed left-sided hemiparesis. The extraoral examination was normal. The intraoral examination revealed the presence of generalized periodontitis. There was no associated teeth mobility. The rest of the airway examination was normal. There was no malocclusion or loose or bucked teeth.

**Figure 1:**
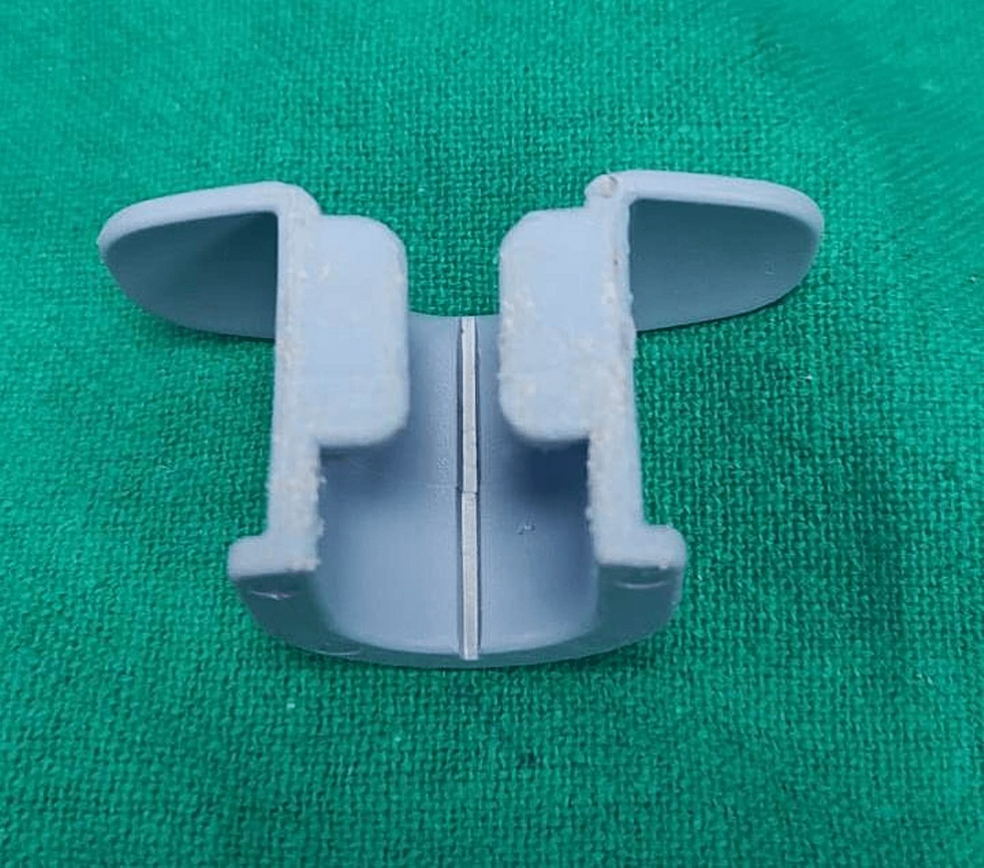
Hard bite block used with the transesophageal echocardiography probe

In the operating room (OR), anesthesia was induced with intravenous fentanyl 100 mcg, propofol 130 mg, and rocuronium 50 mg. Direct laryngoscopy (modified Cormack-Lehane grade two B) and stylet-assisted intubation were performed with a 7.5 mm internal diameter, cuffed, oral, flexometallic endotracheal tube (ETT) in a single attempt. An adult transesophageal echocardiography (TEE) probe was inserted and fixed with a 33 cm mark at the level of the lips. A hard bite block (Figure 1) with gauze wrapped around it was placed around the TEE probe, between the incisors, to secure the TEE probe.

**Figure 2:**
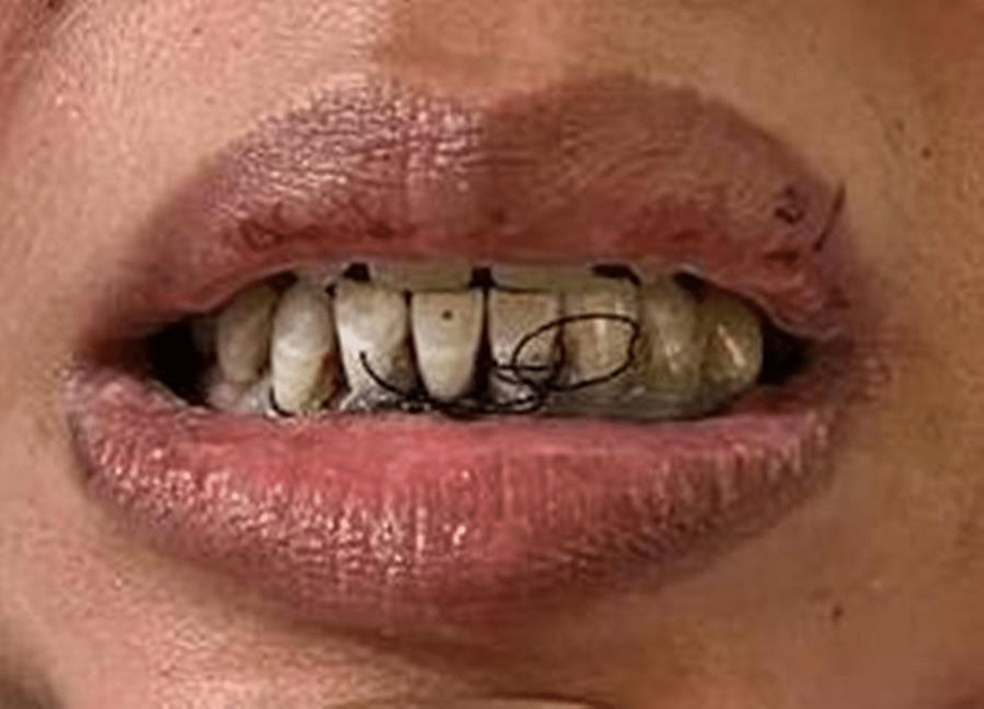
Lower incisors stabilized with suture

A soft bite block made using rolled-up gauze was also placed beside the hard bite block of the TEE probe to prevent tongue injury due to neuromonitoring. Electrodes for MEP and somatosensory-evoked potential (SSEP) monitoring were placed by the neurophysiologist. The patient was then carefully positioned into a sitting position and the neck was flexed with a two-finger breadth distance between the mentum and sternum at the final positioning. Intraoperatively, repeated MEP monitoring was done using 300V stimuli for 75 microseconds comprising 8 pulses in a minute; SSEP monitoring was done using 20-30 mA current and 4.1Hz frequency at 200 microseconds pulse for 100 sweeps (in a span of 20 seconds). Anesthesia was maintained using total intravenous anesthesia (TIVA) with propofol via target-controlled infusion (TCI), dexmedetomidine infusion, and oxygen (50%) with medical air. An end-tidal carbon dioxide concentration of 28-30 mmHg was maintained. The intraoperative period was uneventful and lasted for six hours. Due to surgical handling of the brainstem, the patient was planned for overnight sedation and ventilation. At the end of the surgery, after careful removal of the bite blocks, the lower three incisors were found to be subluxated and displaced forward with surrounding hematoma. The TEE probe was removed carefully. The subluxated teeth were temporarily stabilized in the OR with an Ethilon 2-0 suture by the anesthesiologist (Figure 2), and lignocaine-soaked cotton gauze was placed around the teeth to prevent further bleeding.

No other oropharyngeal injuries were noted. Since the patient was planned for postoperative ventilation, the flexometallic ETT was exchanged for a normal ETT over Cook’s airway exchange catheter. In the neurosurgical intensive care unit (ICU), dental consultation was sought. Oral suctioning was avoided as advised by the dentist. The patient's party was counseled about the injury postoperatively. The rest of the postoperative course was uneventful, and the patient was subsequently discharged on the sixth postoperative day.

## Discussion

A perioperative dental injury is a concerning complication for the patient as well as the anesthetist. In addition to oral soft tissue injuries, tooth enamel fractures and loosening or subluxation of teeth are the most common dental injuries reported perioperatively [2]. Although teeth injuries are more common during intubation, they can occur at any time intraoperatively. Common predisposing factors include the use of undue force during laryngoscopy, carious teeth, buck teeth, teeth with large restorations, inter incisor gap of less than 5 cm, insertion or removal of oropharyngeal or supraglottic airway devices, pre-existing periodontitis, mixed dentition in children, etc. [4-6].

A laryngoscopy-related teeth injury is more likely to affect anterior dentition than posterior ones. Maxillary central incisors, which are sometimes inadvertently used as a fulcrum during laryngoscopy, are even more prone to injury. An anticipated difficult airway increases the risk of dental injury by threefold, while the risk is sevenfold higher with a modified Cormack-Lehane score of three or more [4]. Intubation in our patient was smooth and non-traumatic and was performed in a single attempt. No teeth injury was noticed at intubation.

Endoscopic procedures, transesophageal echocardiography, epilepsy surgery, transcranial MEP monitoring, etc. also can cause dental injuries [7,8]. In our patient, a TEE probe was inserted to monitor for venous air embolism. Intraoperative TEE probe insertion and bite block placement should be a gentle procedure and should be performed preferably under direct laryngoscopic view to reduce the incidence of probe misplacement, multiple attempts at insertion, and associated dental trauma. In our patient, at the time of TEE probe insertion and bite block placement, no injury to teeth or any other oropharyngeal structures was noted. To secure the TEE probe, we placed a hard bite block between the incisors with cotton gauze wrapped around it. Conventional bite blocks used with TEE probes are made of hard, non-pliable plastic material and can be a reason for oropharyngeal injuries. Recently, soft silicone or rubber bite blocks and custom-made mouth guards came into use, which offers the advantage of a comparatively lower incidence of oropharyngeal injury [9]. Soft bite blocks may not completely eliminate dental trauma, but they have been shown to significantly reduce the risk. These soft bite blocks have grown in popularity, as they are a convenient and safe alternative to hand-rolled cotton gauzes and to hard bite blocks, which can cause dental trauma in patients. There is no consensus on the number or type of bite blocks that can prevent oropharyngeal injuries. Some authors have suggested using three soft bite blocks (one in the center and two between the molars on each side) [10]. Displacement of bite blocks during positioning or stimulation for neuromonitoring increases the risk of oropharyngeal injuries. Frequent intraoperative checking of the position of the bite block and tongue must be done, wherever possible. In our patient, the bite blocks were in the proper position till the end of surgery.

Our patient was undergoing surgery in a sitting position with neck flexion. The sitting position with extreme neck flexion is known to be causative of airway edema and tongue injury [11]. However, teeth injuries due to sitting position alone are unlikely. Several instances of oropharyngeal bite injuries and rare cases of dental fractures have been reported with prolonged use of MEP monitoring [8]. MEP monitoring using the intraoperative nerve monitoring system NIM-Eclipse® device (Medtronic Xomed, Minnesota, USA) with the above-mentioned stimulus strengths was performed in our case. We speculate that since our patient was not paralyzed, frequent MEP monitoring with the neck in the flexed position probably resulted in the mandible contracting against the hard TEE probe bite block, resulting in injury to the mandibular incisors. The lower jaw probably did not have enough space to contract during MEP monitoring due to the flexed position of the neck. The presence of pre-existing periodontitis could have further contributed to this injury. The use of less frequent and low voltage stimulation might reduce the risk of such injury as was seen in our case. Earlier authors have also pointed out that the NIM-Eclipse device has a higher incidence of bite injuries compared with other machines. The explanation offered is that this may be related to high current, high voltage capacity, and the use of biphasic stimulus pulses with the NIM-Eclipse machine, which is currently not available for transcranial stimulation on other machines [8].

The use of partial neuromuscular blockade to reduce muscle contractions during stimulation is sometimes used as a strategy to reduce the risk of bite injury [12]. However, this may compromise the quality and interpretability of the MEP responses. Hence, the use of low-dose neuromuscular blocking drugs is not a routine practice to reduce the risk of bite injuries.

We suggest that preoperative consent explaining specific risks involved in intraoperative neuromonitoring can benefit the entire team. The physician must explain the procedure and technique of neuromonitoring and associated risks like oropharyngeal bite injuries, lacerations, and swelling, as well as dental injuries, patient movement-induced injury, seizure, arrhythmias, skin burns, temporo-mandibular joint dislocation, mandible fracture. etc. [13]. A thorough preoperative explanation of expected, although uncommon, complications can go a long way in terms of patient satisfaction.

Another learning point from this experience was that a preoperative dental evaluation in view of pre-existing periodontitis could have avoided this injury. A dental evaluation may not be needed in routine cases. But, in a patient undergoing surgery in a sitting position with neck flexion, with TEE and MEP monitoring and pre-existing periodontitis, a preoperative dental workup seemed warranted. An additional but not less important point is managing complications when they occur. It is uncommon to treat avulsed teeth with an Ethilon 2-0 suture, but in the absence of a dentist, this seemed to be the best management for the anesthesiologist since the patient was in the OR.

## Conclusions

A perioperative, unanticipated dental injury is a distressing complication for both the patient and the anesthesiologist. In patients with a multitude of risk factors for such injuries, as in our patient, preoperative consent regarding the complications associated with neuromonitoring and a thorough preoperative dental workup might prove beneficial. The use of soft silicone-based oral bite blocks and the use of less frequent and low-voltage stimulation may be needed in such patients. The mouth should be inspected to ensure that the tongue and lips are clear of the teeth and not trapped by the bite block. The bite blocks and tongue should be periodically inspected intraoperatively, especially after changes in patient position are made. Also, professional management of intraoperative dental injuries is important.
